# P-446. Outcomes of Hospitalization due to Cryptococcal Meningitis in a High-Resource Setting, 2015-2022

**DOI:** 10.1093/ofid/ofae631.646

**Published:** 2025-01-29

**Authors:** Kirk B Fetters, James C Ziegenbein, Erica Chow, Timothy Hatlen

**Affiliations:** Harbor-UCLA Medical Center, Denver, Colorado; Harbor UCLA, Torrance, California; Harbor-UCLA, Los Angeles, California; Harbor-UCLA Medical Center, Denver, Colorado

## Abstract

**Background:**

There is a paucity of outcome data in high-resource settings for cryptococcal meningitis (CM) in persons living with HIV (PWH). Novel induction regimens, such as the first-line recommended regimen from the World Health Organization, may reduce the duration of hospitalization and drug-associated adverse events with similar treatment outcomes. However, these regimens were not compared to the standard of care used in high-resource countries, leading to a reluctance to change current practice. Randomized controlled trials to compare these strategies would be cost-prohibitive in high-resource settings. To work towards quality observational data on this subject, we sought to describe medication-related adverse effects (AE) and mortality of PWH hospitalized with CM at our institution.

**Table 1:**
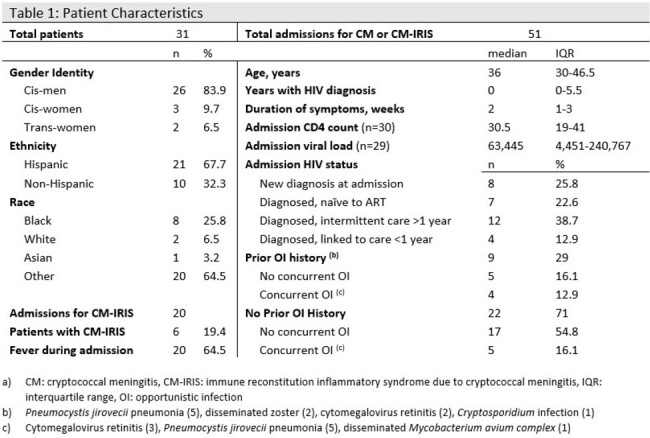
Patient Characteristics

**Methods:**

We retrospectively obtained all electronic medical records associated with inpatient encounters and an HIV diagnosis occurring between 2015-2022 at Harbor-UCLA Medical Center in Los Angeles County, California. We screened for discharge diagnoses of CM or the immune reconstitution inflammatory syndrome due to CM (CM-IRIS). We abstracted demographic, diagnostic, therapeutic, and outcome data from these patient records.

**Table 2:**
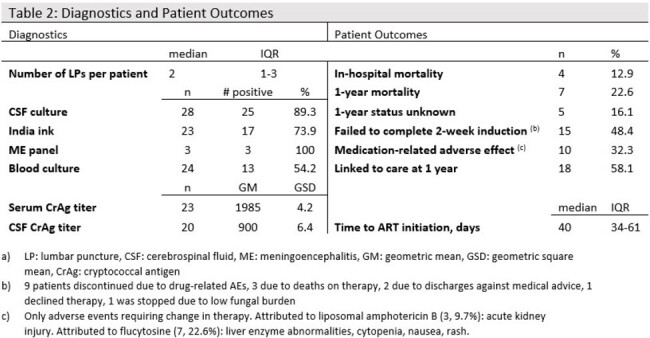
Diagnostics and Patient Outcomes

**Results:**

Thirty-one PWH were hospitalized with CM and/or CM-IRIS for a total of 51 admissions. Of these, 26 (83.9%) were cisgender men, 3 (9.7%) were cisgender women, and 2 (6.5%) were transgender women. The median age was 36 years old (IQR 30-46), and 21 (67.7%) were of self-identified Hispanic ethnicity. The median time since diagnosis of HIV was < 1 year (IQR 0-5.5). All patients were prescribed liposomal amphotericin B at 3-4mg/kg/day and flucytosine (5-FU) 100mg/kg/day for initial treatment. Fifteen (48.4%) patients failed to complete the 2-week induction, 10 (32.3%) due to AE. In-hospital mortality occurred in 4 (12.9%) patients, and 1-year mortality was documented for 7 patients (22.6%). Five patients (16.1%) had unknown status at 1 year.

**Conclusion:**

We report a 1-year mortality of 22% in PWH hospitalized for CM in a high-resource setting compared to about 30-40% reported globally. However, 48% of patients were unable to complete induction, 32% due to adverse drug effects, emphasizing the importance of investigating the effectiveness of novel approaches to therapy.

**Disclosures:**

**All Authors**: No reported disclosures

